# The influence of stimulus onset asynchrony, task order, sex and hormonal contraception on prepulse inhibition and prepulse facilitation: Methodological considerations for drug and imaging research

**DOI:** 10.1177/02698811221133469

**Published:** 2022-10-21

**Authors:** Laura F Naysmith, Steven C R Williams, Veena Kumari

**Affiliations:** 1 Centre for Neuroimaging Sciences, Institute of Psychiatry, Psychology and Neuroscience, King’s College London, London, UK; 2 Department of Psychology, Institute of Psychiatry, Psychology and Neuroscience, King’s College London, London, UK; 3 Centre for Cognitive Neuroscience, College of Health, Medicine and Life Sciences, Brunel University London, London, UK

**Keywords:** Startle reflex, prepulse paradigm, prepulse inhibition, prepulse facilitation, neuropsychopharmacology

## Abstract

**Background:**

Prepulse-induced startle modulation occurs when a weak sensory stimulus (‘prepulse’) is presented before a startling sensory stimulus (‘pulse’), producing an inhibited (Prepulse Inhibition, PPI) or facilitated (Prepulse Facilitation, PPF) startle response. We recently identified several gaps and outlined future lines of enquiry to enable a fuller understanding of the neurobiology of PPI and PPF in healthy and clinical populations. However, before embarking on these studies, it is important to consider how task and population characteristics affect these phenomena in healthy humans.

**Methods:**

We examined PPI and PPF in separate tasks, with counterbalanced task order across participants in one session, using a range of stimulus onset asynchronies (SOAs), in 48 healthy adults (23 men, 25 women; 10 hormonal contraceptive users) to determine which SOAs produce the strongest PPI and PPF and also explored how sex and hormonal contraception might influence PPI and PPF under these experimental conditions.

**Results:**

Both PPI and PPF were affected by SOA, with greatest PPI observed at 60 and 120 ms, and greatest PPF at 4500 and 6000 ms. PPI was influenced by sex (more PPI in men than women) and hormonal contraception, whereas PPF was affected by task order (greater PPF when the PPF task followed, rather than preceded, the PPI task).

**Conclusions:**

Our findings indicate that studies of PPI and PPF need to consider, not only sex and hormonal status of study participants, but also task characteristics and presentation order to reduce variance and increase replicability across studies.

## Introduction

Prepulse-pulse pairing, whereby a weak sensory stimulus (‘prepulse’) precedes a startling sensory stimulus (‘pulse’), is a robust method to modify the startle reflex response. The stimulus onset asynchrony (SOA, i.e., the time between prepulse and pulse) can inhibit, facilitate, or have no effect on the startle response. In healthy humans, the estimated temporal window for inhibiting the startle reflex (Prepulse Inhibition, PPI) is 30–500 ms (Graham, 1975), whereas 500–6000 ms facilitates the startle reflex (Prepulse Facilitation, PPF) (Conzelmann et al., 2010). PPI is theorised to be an operational measure of sensorimotor gating (Braff and Geyer, 1990), providing a neurophysiological index of impaired sensorimotor gating in schizophrenia (Hammer et al., 2011; Swerdlow et al., 2014), Tourette syndrome (Castellanos et al., 1996; Zebardast et al., 2013), post-traumatic stress disorder (Pineles et al., 2016) and several other disorders (review, Swerdlow et al., 2016). PPF is thought to reflect sustained attention and orienting attention (Conzelmann et al., 2010). Clinical studies have shown less PPF in patients with schizophrenia, compared to healthy controls (e.g.,Kumari et al., 2004; Storozheva et al., 2011; Wynn et al., 2004). Thus, prepulse-induced startle modulation is a promising avenue for translational research.

There is an emerging body of literature outlining the neural mechanisms of PPI and PPF, namely thalamic, striatal and frontal activation during PPI in healthy populations (mostly or exclusively males investigated), in addition to activation deficits in these regions in schizophrenia and Tourette syndrome; and superior medial gyrus, cingulate cortex and frontal activation during PPF (Naysmith et al., 2021). As highlighted by Naysmith et al. (2021), gaps in the field include limited PPF research, particularly on the neurobiology of sex differences in PPI and PPF and limited clinical research involving PPF. Understanding the neurobiology of PPI and PPF is essential but, before this can be done, researchers must establish replicable experimental protocols where methodological considerations are made, as task and populations characteristics can impact PPI and PPF. This will better inform drug and imaging studies of PPI/F.

Firstly, the effect of SOA on PPI and PPF needs to be systematically explored. Kumari et al. (2010) used prepulse-pulse trials with SOAs of 30, 60, 120, 240 and 480 ms to study PPI in healthy women at different menstrual cycle phases. SOAs of 120 ms produced the greatest PPI response, which is consistent with existing research, demonstrating up to an 80% decrease in the startle response in healthy volunteers (Heidinger et al., 2019; Lei et al., 2021; Meyhofer et al., 2019). There is, however, more variability in the PPF literature, with studies using a range of SOAs (1000–6000 ms) (Berryman et al., 2021; Kedzior and Martin-Iverson, 2006; Stachtea et al., 2020). Moreover, these SOAs do not always produce facilitatory effects, even within the same study samples (Hong et al., 2008).

Secondly, sex differences in acoustic prepulse-induced startle modulation have been widely reported, since the first observation by Swerdlow and colleagues (Swerdlow et al., 1993), with males showing more PPI than females, and females showing more PPF than males (review, Kumari, 2011). Sex differences in PPI have also been noted in 8-year-old children (Ornitz et al., 1991). Sexual dimorphism in PPI and PPF has been theorised to be explained by the influence of sex hormones on the neurobiology of PPI and PPF, such as on PPI/F neural circuitry (Swerdlow et al., 2001a), and genetics (Quednow et al., 2018). For example, catechol-*O*-methyltransferase (COMT, rs4680), a gene polymorphism associated with PPI (Roussos et al., 2008), has shown sex-specific effects on PPI (Quednow et al., 2018).

The current study aimed to investigate methodological factors, namely SOAs and PPI and PPF task order (when assessed in separate experiments), which might influence acoustic PPI and PPF in a healthy adult sample. Assessment of PPI and PPF in separate tasks may be beneficial, for example, when one is of more interest than the other, such as mapping the neural correlates of PPI/F, or allowing for more exploration of PPF, where inconsistencies have been observed (Hong et al., 2008). This can also optimise certain research studies by reducing the length of testing sessions, for example, when examining the influence of drugs with a short peak response window, such as nicotine, on PPI/F (Baschnagel and Hawk, 2008).

Five different SOAs were used to elicit PPI in one task, and five different SOAs to elicit PPF in another task. These SOAs are commonly used stimulus configurations (e.g., Kumari et al., 2004). The study aimed to establish the effect of SOA on prepulse-induced startle modulation, also identifying which SOA elicits the greatest PPI response, defined as the largest decrease in percentage change of the eyeblink response on prepulse-pulse trials, compared to pulse-only trials; and the greatest PPF response, defined as the largest increase in percentage change in eyeblink response on prepulse-pulse trials, compared to pulse-only trials. PPI- and PPF-related sex differences were investigated. Incidentally, the influence of hormonal contraception on PPI/F was also explored in the sample, with groups composed of men, women on hormonal contraception and women not on hormonal contraception. The five SOAs in the PPI task were all expected to inhibit the startle response, with significant differences expected between temporally distinct SOAs in the PPI response. Similarly, the five SOAs in the PPF task were all expected to facilitate the startle response, with significant differences expected between distinct SOAs in the PPF response. The greatest PPI response was expected at 120 ms, supporting existing findings (Blumenthal and Gescheider, 1987; Hazlett and Buchsbaum, 2001; Kumari et al., 2010), and the greatest PPF response was expected at ∼4500 ms SOA (Kumari et al., 2008a, 2010). Furthermore, we hypothesised that men would show more PPI than women, and women would show more PPF than men. Possible influences of hormonal contraceptive use on PPI/F in women were also explored.

## Methods

The study was approved by the Psychiatry, Nursing and Midwifery Research ethics committee, King’s College London (LRS/DP-20/21-22707).

### Participants

Forty-eight participants (25 female) aged 18–40 years (*M* = 25.15 years, SD = 4.73) took part after meeting inclusion criteria of good health, normal hearing and no history of neurological/psychiatric illness (Table 1). Originally, 56 volunteers were recruited, but eight (six female) were removed from the analysis because of small/non-measurable startle responses (<70% response probability on pulse-only trials, or <60% response probability on prepulse-pulse trials). Where applicable, information was collected on hormonal contraception, menstrual cycle status (first day of the last period) and cycle regularity.

**Table 1.** Demographic characteristics of study participants.

Group	Mean (SD) age in years	Menstrual phase at the date of participation	Contraception type	Self-reported regularity of menstrual cycle
Men (*n* = 23)	25.48 (4.81)	–	–	–
Women (*n* = 25)	24.76 (4.64)	–	–	–
Women on hormonal contraception (*n* = 10)	25.80 (4.42)	*n* = 2 follicular (day 1–13) *n* = 6 luteal (day 15–28) *n* = 2 no menstrual cycle/bleeding	*n* = 7 combined oral contraceptive pill *n* = 1 progesterone-only contraceptive pill *n* = 1 oestrogen-only intrauterine system *n* = 1 progesterone-only intrauterine device	*n* = 7 with regular menstrual cycle *n* = 1 with irregular menstrual cycle *n* = 2 with no menstrual cycle/bleeding
Women not on hormonal contraception (*n* = 15)	24.07 (4.80)	*n* = 8 follicular (day 1–13) *n* = 7 luteal (day 15–28)	–	*n* = 13 with regular menstrual cycle *n* = 2 with irregular menstrual cycle
Total (*n* = 48)	25.15 (4.73)	–	–	–

### PPI/F assessment: procedure and paradigm

Participants were seated in an armchair in a moderately lit laboratory room and were told that the experiment was to measure their response to auditory clicks. No instructions were given as to attend or ignore them. Participants were requested to stay relaxed but keep their eyes open during the experiment. Electrodes were attached by the experimenter (Section ‘Electromyography’) and over-ear headphones were provided. The PPI and PPF listening tasks were presented in a counterbalanced order across participants (PPI–PPF: 24 participants, 12 female; PPF–PPI: 24 participants, 13 female).

The auditory stimuli consisted of a pulse stimulus (40 ms, white noise, 115 dB) and a prepulse stimulus (20 ms, white noise, 85 dB), which were presented over continuous white noise (70 dB). The stimuli were either presented alone or in combination (prepulse-pulse trials).

The PPI task had 46 trials in total. The first four were pulse-only trials (trial one was not included in any analysis), and the remaining trials were arranged into two blocks of 21 trials, with each block consisting of three pulse-only trials, three prepulse-only trials and 15 prepulse-pulse trials with SOAs of 30, 60, 120, 240 and 480 ms (three trials per SOA). The PPF task was identical to the PPI task and also consisted of 46 trials, with the first four trials as pulse-only trials (trial one was not included in any analysis) and the remaining trials arranged into two blocks of 21 trials. Each block consisted of three pulse-only trials, three prepulse-only trials and 15 prepulse-pulse trials with SOAs of 1000, 2000, 3000, 4500 and 6000 ms (three trials per SOA). In both tasks, the trials were pseudo-randomly ordered to ensure that no trial type was repeated in a sequence and the trials were presented to all participants in the same order with inter-stimulus intervals ranging from 9 to 21 s (*M* = 15 s). Both tasks started with an acclimatisation period of 2 min (70 dB, continuous white noise).

### Electromyography

Electromyography (EMG) measured the eyeblink as the startle reflex. Two (contact area <4 mm) Ag/AgCl miniature electrodes, filled with high-conducting electrode gel (SLE, Croydon, UK), were applied to the right orbicularis oculi muscle; the first electrode was 1 cm lateral to and 0.5 cm below the lateral canthus of the participant’s right eye, the second electrode was 1.5 cm below and slightly medial to the latter electrode to be equidistant from the eye. A ground electrode was placed behind the right ear.

A commercially available startle response monitoring system for humans (Mark II, SR-Lab, San Diego, California) delivered the stimuli and recorded EMG activity with a band-pass filter (high-pass 50 Hz and low-pass 1000 Hz), prior to digitising. A notch filter was used to eliminate 60 Hz interference. EMG activity was recorded for 250 ms from the onset of the stimulus, with a sampling interval of 1 ms. EMG data were scored offline using the analytic programme of this system for response amplitude (analogue-to-digital units, A/D) and baseline EMG (the average of the minimum and maximum values recorded from the first 18 ms for each stimulus). Average eyeblink amplitude was a rolling average routine which smoothed the rectified EMG response. Latency to response peak was defined as the latency to the point of maximal amplitude that occurred within 20–100 ms from the onset of startle stimuli. Prior to data scoring, EMG response to each pulse stimulus was reviewed, and any trial with evidence of ongoing blinks before onset of the pulse was excluded. Scoring criteria were identical to those reported previously (Kumari et al., 2008b, 2010, 2012).

### Statistical analysis

Analysis was conducted using SPSS (version 26) and applied an alpha level for significance testing at *p* < 0.05.

Amplitude and habituation of the startle response over the pulse-only trials were examined (separately) for the PPI and PPF tasks, first using a 2 (Sex: men, women) × 2 (Order: PPI–PPF, PPF–PPI) × 3 (Block; three blocks consisting of three pulse-only trials) analysis of variance (ANOVA). Differential amplitude and habituation of the startle response due to hormonal contraception was also explored separately for PPI and PPF using a 3 (Group: men, women on hormonal contraception, women not on hormonal contraception) × 2 (Order) × 3 (Block) ANOVA.

PPI/F was computed for each participant separately for each SOA as (a − b/a) × 100, ‘a’ = pulse-only amplitude and ‘b’ = amplitude over prepulse-pulse trials. Percentage, rather than absolute amount of PPI/F (i.e., arithmetic difference between pulse-only and prepulse-pulse trials), was used to minimise the influence of individual differences in startle responsiveness. Expected sex differences in PPI (SOAs of 30, 60, 120, 240 and 480 ms) and PPF (SOAs of 1000, 2000, 3000, 4500 and 6000 ms) were examined (separately) using a 2 (Sex) × 2 (Order) × 5 (SOA) ANOVA. Hormonal contraception-related PPI and PPF differences were then explored (separately) using a 3 (Group) × 2 (Order) × 5 (SOA) ANOVA.

Startle latency to response peak on the PPI and PPF tasks was examined, first using a 2 (Sex) × 2 (Order) × 6 (Trial type: pulse-only trials and prepulse-pulse trials with 5 SOAs) ANOVA, and then a 3 (Group) × 2 (Order) × 6 (Trial type) ANOVA.

For all ANOVAs described earlier, significant interaction or main effects were followed up by lower order ANOVAs and the analysis of simple main effects using *t*-tests. Repeated measures with more than two levels employed the Greenhouse–Geisser epsilon (ε) correction.

## Results

### Startle amplitude and habituation

The Sex × Order × Block ANOVA (PPI task) revealed a main effect of Block (*F* = 26.507, df = 2, 88, *p* < 0.001), but not Sex or Order (Supplemental Appendix A), showing habituation of response to pulse-only trials over the task with significantly greater response amplitude on Block 1, compared to Blocks 2 (*t* = 4.95, df = 47, *p* < 0.001) and 3 (*t* = 6.35, df = 47, *p* < 0.001) (Table 2). Response amplitude did not differ significantly between Blocks 2 and 3. An interaction between Block × Order (*F* = 6.327, df = 2, 44, *p* = 0.003) was also found, indicating significant differences between participants assigned to the two Orders across all Blocks (Block 1: *t* = 12.252, df = 47, *p* < 0.001; Block 2: *t* = 9.200, df = 47, *p* < 0.001; Block 3: *t* = 9.175, df = 47, *p* < 0.001), with greater response amplitude in participants for whom the PPI task was presented first, rather than second (Block 1_first_ = 784.52 A/D, Block 1_second_ = 609.98 A/D; Block 2_first_ = 573.08 A/D, Block 2_second_ = 524.40 A/D; Block 3_first_ = 541.30 A/D, Block 3_second_ = 515.67 A/D). Other interactions between Sex, Order and Block were non-significant (Supplemental Appendix A).

**Table 2.** Mean (standard error of the mean, SEM) startle amplitudes (A/D) over pulse-only trials and mean (SEM) PPI and PPF (%) for all participants and groups.

Task	Measure	Overall mean (SEM)	Men mean (SEM)	Women mean (SEM)	Women on hormonal contraception mean (SEM)	Women not on hormonal contraception mean (SEM)
PPI	Amplitude on pulse-only trials [A/D]					
	Block 1	652.34 (53.26)	597.64 (77.16)	707.04 (73.44)	809.91 (127.04)	684.20 (97.03)
	Block 2	496.19 (54.78)	404.02 (79.36)	588.36 (75.53)	687.13 (131.61)	555.07 (100.52)
	Block 3	459.71 (50.18)	360.41 (72.70)	559.00 (69.19)	722.06 (117.16)	502.98 (89.48)
	PPI [%]					
	SOA 30 ms	24.42 (2.98)	31.19 (4.29)	17.65 (4.11)	17.81 (7.04)	14.41 (5.38)
	SOA 60 ms	44.40 (3.65)	51.30 (5.18)	37.50 (5.03)	48.80 (8.49)	29.21 (6.48)
	SOA 120 ms	44.06 (4.63)	53.63 (6.63)	34.50 (6.38)	42.00 (10.88)	26.27 (8.31)
	SOA 240 ms	35.71 (4.22)	47.43 (6.04)	23.99 (5.82)	22.89 (9.90)	19.97 (7.56)
	SOA 480 ms	23.40 (4.14)	32.91 (5.84)	13.89 (5.71)	6.92 (9.59)	12.47 (7.32)
PPF	Amplitude on pulse-only trials [A/D]					
	Block 1	704.90 (59.98)	606.26 (84.98)	803.54 (83.06)	995.29 (141.77)	718.15 (108.28)
	Block 2	507.22 (49.44)	408.74 (70.04)	605.69 (68.44)	740.56 (117.64)	554.23 (89.85)
	Block 3	463.32 (50.45)	364.88 (71.48)	375.43 (71.23)	644.05 (121.51)	496.21 (92.81)
	PPF [%]					
	SOA 1000 ms	1.29 (3.44)	4.66 (4.96)	−2.08 (4.76)	−8.82 (8.17)	−1.22 (6.24)
	SOA 2000 ms	3.22 (3.35)	7.21 (4.84)	−0.77 (4.64)	−3.20 (8.13)	−1.00 (6.21)
	SOA 3000 ms	−4.83 (3.19)	−0.24 (4.61)	−9.41 (4.42)	−11.73 (7.78)	−8.08 (5.94)
	SOA 4500 ms	−8.71 (3.69)	−6.71 (5.33)	−10.72 (5.11)	−13.39 (9.01)	−9.23 (6.88)
	SOA 6000 ms	−12.41 (3.31)	−13.56 (4.78)	−11.25 (4.58)	−16.29 (7.91)	−11.14 (6.04)

The Group × Order × Block ANOVA (PPI task) to explore hormonal contraception-related differences in startle amplitude and habituation over pulse-only trials also revealed a main effect of Block (*F* = 17.164, df = 2, 84, corrected *p* < 0.001, ε = 0.947; Table 2), but not Group (*F* = 2.194, df = 2, 42, *p* = 0.124) or Order (*F* < 1, df = 1, 42, *p* = 0.460). Here, there was also a significant interaction between Block × Order (*F* = 3.246, df = 2, 84, *p* = 0.044), with all other interactions being non-significant (all *p* > 0.370).

The Sex × Order × Block ANOVA (PPF task) revealed a main effect of Block (*F* = 33.200, df = 2, 88, *p* < 0.001), but not Sex, Order, or any interaction effects (Supplemental Appendix A). The effect of Block highlighted habituation of response to pulse-only trials, with significantly lower response amplitude in Block 2 versus 1 (*t* = 6.401, df = 47, *p* < 0.001) and Block 3 versus 1 (*t* = 6.925, df = 47, *p* < 0.001) (Table 2). Response amplitude did not differ between Blocks 2 and 3 (*t* = 1.559, df = 47, *p* = 0.126).

The Group × Order × Block ANOVA (PPF task) to examine startle amplitude and habituation to pulse-only trials confirmed response habituation over the blocks of pulse-only trials as described above, revealing a main effect of Block (*F* = 34.102, df = 2, 84, *p* < 0.001; Table 2) but not Group (*F* = 2.886, df = 2, 42, *p* = 0.067), Order (*F* = 2.307, df = 1, 42, *p* = 0.136) or interaction effects (all *p* > 0.120).

### Prepulse inhibition

A Sex × Order × SOA ANOVA revealed a main effect of SOA (*F* = 19.556, df = 2.883, 126.848, corrected *p* < 0.001, ε = 0.721; Table 2) and Sex (*F* = 6.900, df = 1, 44, *p* = 0.012), but not Order or any interactions involving Sex, Order and SOA (Supplemental Appendix A). The main effect of SOA revealed greatest PPI at 60 and 120 ms, with no significant difference between these two SOAs (*t* = 0.062, df = 47, *p* = 0.951). PPI was lower and comparable at 480 and 30 ms (*t* = 0.214, df = 47, *p* = 0.831), thus illustrating an inhibitory curve over all SOAs. The main effect of Sex revealed significantly greater PPI in men (*M* = 43.29%), compared to women (*M* = 25.51%).

**Figure 1.**
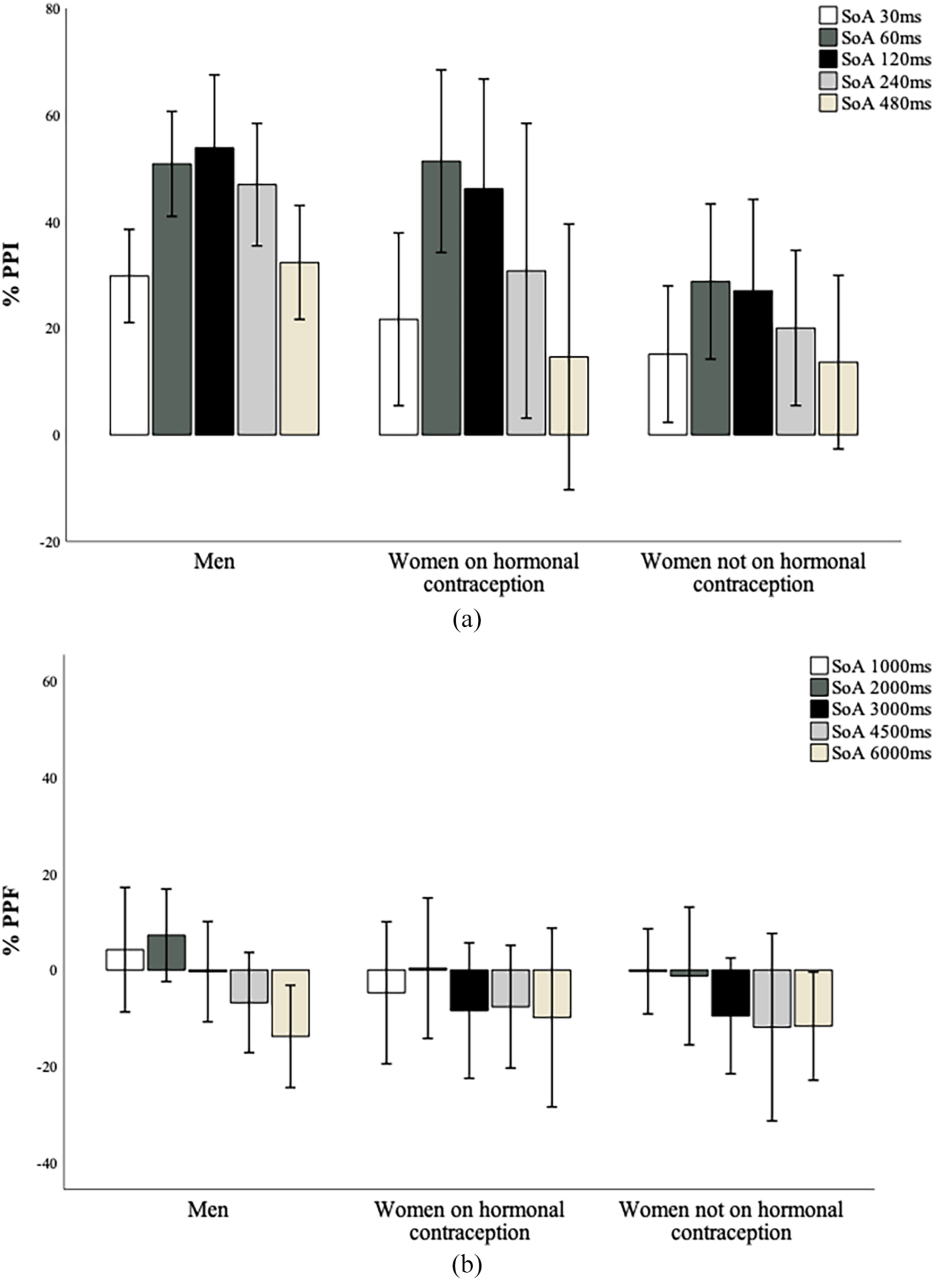
(a) Mean PPI (%) across groups. Positive values represent response inhibition. Error bars show ±1 SE of the mean. *p < 0.05; men showed significantly greater PPI across all SOAs, compared to women not on hormonal contraception. (b) Mean PPF (%) across groups. Negative values represent response facilitation. Error bars show ±1 SE of the mean. No significant group differences in PPF across SOAs.

A Group × Order × SOA ANOVA to explore hormonal contraception-related differences in PPI also revealed a main effect of SOA (*F* = 20.325, df = 2.906, 122.072, corrected *p* < 0.001, ε = 0.727), in addition to Group (*F* = 4.669, df = 2, 42, *p* = 0.015; Figure 1(a)), but not Order (*F* = 1.969, df = 1, 42, *p* = 0.168). The main effect of Group revealed that men showed significantly greater PPI than women not on hormonal contraception (*F* = 9.131, df = 1, 36, *p* = 0.005) across all SOAs (30 ms: *t* = 2.066, df = 36, *p* = 0.046; 60 ms: *t* = 2.746, df = 36, *p* = 0.009; 120 ms: *t* = 2.573, df = 36, *p* = 0.024; 240 ms: *t* = 3.062, df = 36, *p* = 0.004; 480 ms: *t* = 2.115, df = 36, *p* = 0.041). There were not significant group differences for men vs women on hormonal contraception (*F* = 1.287, df = 1, 31, *p* = 0.265) or women not on hormonal contraception vs women on hormonal contraception (*F* = 1.329, df = 1, 23, *p* = 0.261). All interactions involving Group, SOA or Order were non-significant (all *p* > 0.130).

### Prepulse facilitation

A Sex × Order × SOA ANOVA revealed a main effect of SOA (*F* = 6.670, df = 3.129, 137.679, corrected *p* < 0.001, ε = 0.782; Table 2) and Order (*F* = 1.029, df = 1, 44, *p* = 0.042; Table 3), but not Sex or any interaction effects involving Sex, Order and SOA (Supplemental Appendix A). The main effect of SOA outlined greatest PPF at 6000 and 4500 ms, where PPF did not differ significantly between the two SOAs (*t* = 1.027, df = 47, *p* = 0.310). PPF at 6000 ms was significantly greater than all remaining SOAs (1000 ms: *t* = 3.812, df = 47, *p* < 0.001; 2000 ms: *t* = 4.291, df = 47, *p* < 0.001; 3000 ms: *t* = 2.303, df = 47, *p* = 0.026). SOAs of 1000 and 2000 ms produced the weakest PPF, even revealing an inhibitory effect, and produced significantly weaker PPF than 3000 ms (*t* = 2.674, df = 47, *p* = 0.010) and 4500 ms (*t* = 3.017, df = 47, *p* = 0.004). Other mean comparisons were non-significant. The Order effect revealed greater PPF across all SOAs when the PPF task was presented second, rather than first (*M*_first_ = 1.00%; *M*_second_ = −9.57%).

**Table 3.** Mean (SEM) PPF (%) across all participants when the PPF task was presented first (PPF–PPI) or second (PPI–PPF).

Order	SOA	Men mean (SEM)	Women mean (SEM)	Women on hormonal contraception mean (SEM)	Women not on hormonal contraception mean (SEM)
**First**	SOA 1000 ms	14.92 (7.17)	−1.95 (6.59)	1.37 (8.95)	−5.82 (9.67)
	SOA 2000 ms	7.44 (6.99)	3.19 (6.43)	5.70 (8.90)	0.27 (9.62)
	SOA 3000 ms	2.49 (6.66)	−2.19 (6.12)	−3.53 (8.53)	−0.63 (9.21)
	SOA 4500 ms	−4.84 (7.70)	2.39 (7.09)	0.94 (9.87)	4.09 (10.66)
	SOA 6000 ms	−7.43 (6.90)	−4.06 (6.35)	−0.30 (8.66)	−8.45 (9.36)
**Second**	SOA 1000 ms	−5.61 (6.86)	−2.22 (6.86)	−19.01 (13.68)	3.38 (7.90)
	SOA 2000 ms	6.98 (6.69)	−4.72 (6.69)	−12.09 (13.60)	−2.27 (7.85)
	SOA 3000 ms	−2.96 (6.37)	−16.63 (6.37)	−19.94 (13.02)	−15.53 (7.52)
	SOA 4500 ms	−8.57 (7.38)	−23.84 (7.38)	−27.72 (15.08)	−22.55 (8.70)
	SOA 6000 ms	−19.70 (6.61)	−18.45 (6.61)	−32.28 (13.23)	−13.84 (7.64)

A Group × Order × SOA ANOVA to explore hormonal contraception-related differences in PPF also showed a main effect of SOA (*F* = 4.248, df = 3.158, 132.655, corrected *p* = 0.006, ε = 0.790; Table 2) and Order (*F* = 5.434, df = 1, 42, *p* = 0.025; Table 3) but not Group (*F* < 1, df = 2, 42, *p* = 0.431; Figure 1(b)). All interactions involving Group, Order and SOA were non-significant (all *p* > 0.210).

### Latency to response peak

The Sex × Order × Trial type ANOVA (PPI task) revealed a main effect of Trial type (*F* = 9.854, df = 3.715, 163.464, corrected *p* < 0.001, ε = 0.743; Table 4), but not Sex or Order (Supplemental Appendix A), with significantly longer response latencies on pulse-only trials, compared to all other trials. In addition, trials of 480 ms had significantly longer response latencies than all other (shorter) SOAs. A significant interaction between Trial type × Sex (*F* = 2.991, df = 3.712, 155.943, corrected *p* = 0.023, ε = 0.743) was observed, revealing significantly longer response latencies in men on trials of 480 ms (*t* = 2.246, df = 46, *p* = 0.030) and pulse-only trials (*t* = 3.446, df = 46, *p* = 0.001). All other independent *t*-tests were non-significant. No other interaction effects between Sex, Order and Trial type were present (Supplemental Appendix A).

**Table 4.** Mean (SEM) latencies (ms) to peak for pulse-only and prepulse-pulse trials during the PPI and PPF tasks.

Task	Trial type	Overall mean (SEM)	Men mean (SEM)	Women mean (SEM)	Women on hormonal contraception mean (SEM)	Women not on hormonal contraception mean (SEM)
Response latency to peak (ms)						
PPI	Pulse-only	63.24 (1.01)	66.64 (1.47)	59.85 (1.40)	62.65 (2.41)	58.32 (1.84)
	SOA 30 ms	54.30 (1.23)	54.68 (1.77)	54.18 (1.69)	52.13 (2.91)	56.08 (2.23)
	SOA 60 ms	53.84 (1.13)	52.34 (1.64)	55.33 (1.56)	54.84 (2.75)	55.58 (2.10)
	SOA 120 ms	55.99 (2.04)	54.93 (2.95)	57.05 (2.81)	58.37 (4.88)	57.87 (3.73)
	SOA 240 ms	56.42 (1.51)	55.37 (2.19)	57.47 (2.08)	60.09 (3.64)	55.89 (2.78)
	SOA 480 ms	60.95 (1.13)	63.54 (1.64)	58.37 (1.56)	60.43 (3.69)	58.13 (2.06)
PPF	Pulse-only	60.97 (0.88)	62.62 (1.28)	59.31 (1.22)	61.10 (2.13)	58.68 (1.62)
	SOA 1000 ms	63.40 (0.92)	66.15 (1.32)	60.66 (1.27)	63.36 (2.17)	59.61 (1.66)
	SOA 2000 ms	62.12 (1.18)	64.22 (1.70)	60.02 (1.63)	62.68 (2.82)	59.23 (2.15)
	SOA 3000 ms	60.48 (0.90)	61.64 (1.30)	59.31 (1.25)	62.52 (2.11)	57.88 (1.61)
	SOA 4500 ms	60.61 (0.81)	62.33 (1.17)	58.55 (1.12)	59.95 (1.93)	58.99 (1.48)
	SOA 6000 ms	61.19 (0.76)	62.79 (1.10)	59.59 (1.06)	61.85 (1.81)	58.54 (1.38)

The Group × Order × Trial type ANOVA on latencies to response peak during the PPI task also revealed a main effect of Trial type (*F* = 6.305, df = 3.712, 155.943, corrected *p* < 0.001, ε = 0.743; Table 4), but no other main or interaction effects were significant (all *p* > 0.160).

The Sex × Order × Trial type ANOVA (PPF task) on latencies to response peak revealed a main effect of Trial type (*F* = 3.715, df = 4.069, 179.030, corrected *p* = 0.006, ε = 0.814; Table 4) and Sex (*F* = 5.954, df = 1, 44, *p* = 0.019), but not Order or any interaction effects involving Sex, Order and Trial type (Supplemental Appendix A). The main effect of Trial type showed significantly longer response latencies on trials of 1000 ms, compared to 3000, 4500, 6000 ms and pulse-only trials, but not 2000 ms (*t* = 1.650, df = 47, *p* = 0.106). Response latencies did not differ between other trials. The main effect of Sex showed significantly longer response latencies in men (*M* = 63.29 ms), compared to women (*M* = 59.63 ms)

The Group × Order × Trial type ANOVA (PPF task) on latencies to response peak revealed main effects of Group (*F* = 3.374, df = 2, 42, *p* = 0.044; Table 4), Order (*F* = 5.359, df = 1, 42, *p* = 0.026) and a marginal effect of Trial type (*F* = 2.317, df = 4.018, 168.766, corrected *p* = 0.054, ε = 0.804) which was in line with the Trial type effects described above. The Group effect revealed that men showed significantly longer response latencies, compared to women not on hormonal contraception (*p* = 0.040); other group comparisons were non-significant. The main effect of Order showed significantly shorter peak latencies on all trial types when the PPF task was presented first, compared to when it was presented second (*M*_first_ = 59.43 ms; *M*_second_ = 63.25 ms). All interaction effects were non-significant (all *p* > 0.160).

## Discussion

In this study of healthy adults using an auditory prepulse paradigm to assess PPI and PPF in separate tasks, it was found that both PPI and PPF were significantly affected by SOA. The greatest PPI was found during SOAs of 60 and 120 ms, and the greatest PPF response at 4500 and 6000 ms SOAs. Importantly, PPF (but not PPI) was significantly affected by task order, as presenting the PPF task after the PPI task led to significantly greater PPF, compared to when the PPF task preceded the PPI task. Men showed greater PPI than women, but PPF was not affected by sex. Our exploratory work suggested that hormonal contraception may influence PPI, as greater PPI was observed in men compared to the subgroup of women who were not on hormonal contraception. No sex differences were found in startle reactivity, although PPI in some studies has been found to be dependent on baseline startle reactivity (Csomor et al., 2008).

As hypothesised, all SOAs on the PPI task produced an inhibited response, with the greatest PPI response at 60 and 120 ms which peaked at 44% across the whole sample. In accord with Kumari et al. (2010), an inhibitory curve was observed over the SOAs; PPI increases, peaks and falls. Furthermore, our findings are supported by the existing literature showing the greatest PPI response on trials with SOAs of 60 and 120 ms (Aasen et al., 2005; Kumari et al., 2010). Here, we further demonstrate that these commonly configured SOAs, 60 and 120 ms, which produce the greatest PPI within a healthy adult sample. There are, of course, many other task manipulations, for example, prepulse intensity or duration, which would also impact PPI/F (Braff et al., 2001), and thus need to be considered.

Our findings regarding PPF varied slightly from our original hypothesis as not all SOAs elicited a facilitated startle response, and the greatest PPF occurred at 6000 ms, and not ∼4500 ms as was the case in previous studies that had combined PPI and PPF trials within the same experiment and used SOAs of 1000–6000 ms to induce PPF in healthy people (Aasen et al., 2005; Kumari et al., 2008, 2010). It is possible that when PPI and PPF trials are presented within a single task, the PPI trials (whereby the prepulse is presented in close proximity to the pulse) somehow impact the PPI–PPF curve. However, further work, preferably including a large range of SOAs and tasks with mixed as well as separate PPI and PPF trials, is required to fully examine this possibility since PPF at 4500 ms in earlier studies often did not significantly differ from PPF at 6000 ms.

Indeed, task order presentation must also be a methodological consideration in PPF research. Across all participants, greater PPF was observed with a PPI–PPF task order, whereas PPF–PPI failed to consistently produce PPF across all SOAs. Inconsistent PPF has similarly been observed in human (Hong et al., 2008) and rodent studies (Sasaki et al., 1998). It is possible that while PPI may get attenuated with habituation over pulse-only trials (Quednow et al., 2006), the opposite is true for PPF, and it will emerge in the second session after exposure to (and habituation over) pulse-only trials, with or without the presence of PPI (prepulse + pulse) trials. Alternatively, PPF may be affected by context, such as task order, in this case. Brymer et al. (2021) showed SOAs of 30 ms did not induce PPF in rats who had corticosterone injections, whereas vehicle rats showed PPF. Repeated corticosterone injections caused stress on the hippocampus, which has been linked to contextual learning (Davachi and DuBrow, 2015) and consequently affected PPF. Although more work is needed to distinguish between the possibilities mentioned earlier, it does seem that exposure to PPI trials, either within the same experiment or prior to the PPF session, is likely to elicit greater, and more consistent, PPF in healthy humans.

Men showed more PPI than women and one would expect to see sexual dimorphism in PPI (Abel et al., 1998; Ison and Allen, 2007; Koch, 1998; Swerdlow et al., 1993), which has been theoretically linked to sex hormones. Adding further to the role of sex hormones, we provide preliminary findings of PPI differences relating to hormonal contraception use which has not yet been firmly established. Gogos (2013) did not find PPI differences between oral contraceptive users, non-oral contraceptive users, and men. Borgstrom et al. (2008) found lower PPI in combined oral contraceptive users who reported negative mood, in comparison to users who had not reported negative mood, theorising that PPI differences resulted from contraception-related negative affect, rather than contraception use alone. In addition to previous findings that highlight population characteristics which may affect PPI/F, such as menstrual cycle (Jovanovic et al., 2004) and nicotine (Hong et al., 2008), the current study illustrates the need to assess sex and hormonal contraception when designing PPI/F studies. This is important for drug and imaging studies to explore sexual dimorphism on a neurobiological level.

The current study is not without limitations. Firstly, a larger female sample with randomised hormonal contraception type with attention to contraception type, length of use and hormonal status at the time of testing is needed to draw statistically powerful conclusions concerning the influence of hormonal contraception on PPI. Pharmacological and imaging research may then wish to explore the role of oestrogen and progesterone in PPI to examine the role of sex hormones in clinical disorders. Secondly, the effect of age (Ellwanger et al., 2003; Giannopoulos et al., 2022) and nicotine (Baschnagel and Hawk, 2008) was not explored and has previously been shown to affect PPI and PPF. In addition, test-retest reliability of PPF and internal consistency (i.e., how PPI/F varies across blocks; Supplemental Appendix B) should be established to better understand the order effect on PPF and implications for designing PPI/F studies for clinical research.

In conclusion, we observed differences in PPI and PPF relating to SOA, with greatest PPI across all participants at 60 and 120 ms and the greatest PPF response at 4500 and 6000 ms. Significantly greater PPF occurred when the task was presented after the PPI task, rather than before, thus we report the importance of task order when using two separate tasks to investigate PPI and PPF. Men showed significantly greater PPI than women. Incidentally, hormonal contraception-related PPI differences were observed. The current study has highlighted methodological considerations for PPI/F research and has important implications for designing a robust and replicable auditory prepulse task for use in pharmacology and imaging studies based on a healthy adult sample.

## Supplemental Material

sj-docx-1-jop-10.1177_02698811221133469 – Supplemental material for The influence of stimulus onset asynchrony, task order, sex and hormonal contraception on prepulse inhibition and prepulse facilitation: Methodological considerations for drug and imaging research Click here for additional data file. Supplemental material, sj-docx-1-jop-10.1177_02698811221133469 for The influence of stimulus onset asynchrony, task order, sex and hormonal contraception on prepulse inhibition and prepulse facilitation: Methodological considerations for drug and imaging research by Laura F Naysmith, Steven C R Williams and Veena Kumari in Journal of Psychopharmacology

sj-docx-2-jop-10.1177_02698811221133469 – Supplemental material for The influence of stimulus onset asynchrony, task order, sex and hormonal contraception on prepulse inhibition and prepulse facilitation: Methodological considerations for drug and imaging research Click here for additional data file. Supplemental material, sj-docx-2-jop-10.1177_02698811221133469 for The influence of stimulus onset asynchrony, task order, sex and hormonal contraception on prepulse inhibition and prepulse facilitation: Methodological considerations for drug and imaging research by Laura F Naysmith, Steven C R Williams and Veena Kumari in Journal of Psychopharmacology
