# Supplementary material for: The influence of stimulus onset asynchrony, task order, sex and hormonal contraception on prepulse inhibition and prepulse facilitation: Methodological considerations for drug and imaging research
Source: J Psychopharmacol. 2022 Oct 21;36(11):1234–42. doi: 10.1177/02698811221133469 (PMC9643818; doi:10.1177/02698811221133469)
Supplement: sj-docx-1-jop-10.1177_02698811221133469 – Supplemental material for The influence of stimulus onset asynchrony, task order, sex and hormonal contraception on prepulse inhibition and prepulse facilitation: Methodological considerations for drug and imaging research [file sj-docx-1-jop-10.1177_02698811221133469.docx]

**6.0 Supplementary Materials**

**Appendix A**

Table 5. Non-significant (*p*>0.05) results from mixed measures ANOVA for sections 3.1, 3.2 and 3.3.

| Task |  | Degrees of freedom, error | F | p |
| --- | --- | --- | --- | --- |
| *Mixed measures ANOVA results for startle amplitude and habituation (3.1)* | | | | |
| PPI | Sex | 1, 44 | 2.670 | 0.109 |
|  | Order | 1, 44 | 0.378 | 0.542 |
|  | Block * Sex | 2, 88 | 1.453 | 0.239 |
|  | Sex * Order | 1, 44 | 1.037 | 0.314 |
|  | Block * Sex * Order | 2, 88 | 0.953 | 0.390 |
|  |  |  |  |  |
| PPF | Sex | 1, 44 | 3.836 | 0.057 |
|  | Order | 1, 44 | 3.011 | 0.090 |
|  | Block * Sex | 2, 88 | 0.000 | 1.000 |
|  | Sex * Order | 1, 44 | 1.966 | 0.168 |
|  | Block * Order | 2, 88 | 2.593 | 0.081 |
|  | Block * Sex * Order | 2, 88 | 0.210 | 0.811 |
| *Mixed measures ANOVA results for prepulse-induced startle modulation (3.2)* | | | | |
| PPI | Order | 1, 44 | 1.872 | 0.178 |
|  | Block * Sex | 2.883, 126.848^a^ | 0.814 | 0.484 |
|  | Sex * Order | 1, 44 | 0.010 | 0.921 |
|  | Block * Order | 2.883, 126.848^a^ | 0.843 | 0.469 |
|  | Block * Sex * Order | 2.883, 126.848^a^ | 1.593 | 0.196 |
|  |  |  |  |  |
| PPF | Block | 3.129, 137.679^b^ | 6.670 | 0.196 |
|  | Order | 1, 44 | 1.029 | 0.316 |
|  | Sex * Order | 1, 44 | 0.170 | 0.498 |
|  | Block * Order | 3.129, 137.679^b^ | 0.656 | 0.587 |
|  | Block * Sex * Order | 3.129, 137.679^b^ | 2.336 | 0.498 |
| *Mixed measures ANOVA results for latency to response peak (3.3)* | | | | |
| PPI | Sex | 1, 44 | 0.266 | 0.609 |
|  | Order | 1, 44 | 1.724 | 0.196 |
|  | Sex * Order | 1, 44 | 0.030 | 0.864 |
|  | Trial type * Order | 3.715, 163.464^c^ | 1.876 | 0.122 |
|  | Trial type * Sex * Order | 3.715, 163.464^c^ | 1.476 | 0.215 |
|  |  |  |  |  |
| PPF | Order | 1, 44 | 3.602 | 0.064 |
|  | Trial type * Sex | 4.069, 179.030^d^ | 0.867 | 0.486 |
|  | Sex * Order | 1, 44 | 0.003 | 0.953 |
|  | Trial type * Order | 4.069, 179.030^d^ | 0.624 | 0.649 |
|  | Trial type * Sex * Order | 4.069, 179.030^d^ | 0.820 | 0.516 |
