# Supplementary material for: The influence of stimulus onset asynchrony, task order, sex and hormonal contraception on prepulse inhibition and prepulse facilitation: Methodological considerations for drug and imaging research
Source: J Psychopharmacol. 2022 Oct 21;36(11):1234–42. doi: 10.1177/02698811221133469 (PMC9643818; doi:10.1177/02698811221133469)
Supplement: sj-docx-2-jop-10.1177_02698811221133469 – Supplemental material for The influence of stimulus onset asynchrony, task order, sex and hormonal contraception on prepulse inhibition and prepulse facilitation: Methodological considerations for drug and imaging research [file sj-docx-2-jop-10.1177_02698811221133469.docx]

**6.0 Supplementary Materials**

**Appendix B**

Table 6: Correlations (Pearson’s r) between PPI and PPF (%) during the two blocks of trials (Block 1, Block 2) for all participants and groups.

| Task | Measure | Pearson’s R  Overall | Pearson’s R  Men | Pearson’s R  Women | Pearson’s R  Women on hormonal contraception | Pearson’s R  Women not on hormonal contraception |
| --- | --- | --- | --- | --- | --- | --- |
| PPI | SOA 30 ms | -.070 | .336 | .024 | .365 | -.156 |
|  | SOA 60 ms | .363* | .110 | .568** | .601 | .426 |
|  | SOA 120 ms | .521** | .480* | .779** | .802** | .429 |
|  | SOA 240 ms | .580** | .385** | .481* | .851** | .150 |
|  | SOA 480 ms | .294* | .377 | .177 | .643* | -.049 |
|  |  |  |  |  |  |  |
| PPF | SOA 1000 ms | .115 | .074 | .050 | .103 | -.069 |
|  | SOA 2000 ms | .020 | .079 | .112 | .073 | .145 |
|  | SOA 3000 ms | .243 | .198 | -.321 | -.312 | -.261 |
|  | SOA 4500 ms | .053 | .098 | .174 | -.325 | .405 |
|  | SOA 6000 ms | .064 | .027 | -.108 | .323 | -.271 |

* significant at p<0.05

** significant at p<0.01 or less
